# Data on immunogenicity and reactogenicity to COVID-19 vaccination among patients receiving maintenance dialysis

**DOI:** 10.1016/j.dib.2022.108271

**Published:** 2022-05-16

**Authors:** Hristos Karakizlis, Christian Nahrgang, Kevin Strecker, Jiangping Chen, Mostafa Aly, Heiko Slanina, Christian G. Schüttler, Isla Esso, Martin Wolter, Darina Todorova, Sönke Jessen, Andrea Adamik, Claudio Ronco, Werner Seeger, Rolf Weimer, Martina Sester, Horst-Walter Birk, Faeq Husain-Syed

**Affiliations:** a Department of Internal Medicine II, University Hospital Giessen and Marburg, Justus-Liebig-University Giessen, Klinikstraße 33, Giessen 35392, Germany; b AID GmbH, Ebinger Straße 4, Strassberg 72479, Germany; c Transplantation Immunology, Institute of Immunology, University Hospital Heidelberg, Im Neuenheimer Feld 305, Heidelberg 69120, Germany; d Nephrology Unit, Internal Medicine Department, Assiut University, Governorate 71515, Assiut, Egypt; e Institute of Medical Virology, Justus-Liebig-University Giessen, Schubertstraße 81, Giessen 35392, Germany; f Department of Nephrology, Dialysis and Transplantation, International Renal Research Institute of Vicenza, San Bortolo Hospital, Via Rodolfi, Vicenza 37–36100, Italy; g Department of Medicine (DIMED), Università di Padova, Via Giustiniani, Padua 2–35128, Italy; h Member of the German Center for Lung Research (DZL), Universities of Giessen and Marburg Lung Center (UGMLC), Klinikstraße 33, Giessen 35392, Germany; i Department of Lung Development and Remodeling, Max Planck Institute for Heart and Lung Research, Ludwigstraße 43, Bad Nauheim 61231, Germany; j Department of Transplant and Infection Immunology, Saarland University, Kirrberger Straße, Homburg 66421, Germany

**Keywords:** Cellular immune response, Hemodialysis, Immunoglobulins, Peritoneal dialysis, SARS-CoV-2, T cells

## Abstract

Compared with the general population, patients receiving maintenance dialysis are at increased risk for morbidity and mortality associated with coronavirus disease 2019 (COVID-19). Currently, data on severe acute respiratory syndrome coronavirus type 2 (SARS-CoV-2)-specific immunity post-vaccination in patients on maintenance dialysis are scarce given that the effectiveness of the vaccines has not been explicitly tested in this population due to their common exclusion from SARS-CoV-2 vaccination trials. We herein present data of the specific cellular (interferon-γ and interleukin-2 ELISpot assays) and humoral immune responses (dot plot array and chemiluminescent microparticle immunoassay) at 4 weeks and 6 weeks following a single dose or a complete homologous dual dose SARS-CoV-2 vaccine regimen in 60 adult patients on maintenance dialysis (six with a history of COVID-19). The data was produced in a framework of a project focused on a) quantifying the immune response after full vaccination, b) evaluating the short-term durability of immune response, and c) examining the reactogenicity of SARS-CoV-2 vaccine regimens in patients on maintenance dialysis.

## Specifications Table

**Table:** 

Subject	Health and medical sciences: Nephrology
Specific subject area	Chronic kidney disease; kidney failure; SARS-CoV-2; vaccination.
Type of data	Figure
How the data were acquired	The data were acquired via FluoroSpot Immune assay kit, dot plot array, chemiluminescent microparticle immunoassay, and a survey.
Data format	Analyzed Raw
Description of data collection	Blood samples were obtained prior to dialysis treatment at 4 weeks and 6 weeks after complete vaccination. Reactogenicity data were self-reported using a standardized questionnaire. The data were merged from the University Hospital Giessen medical records, the questionnaires, and the immune response data from GenID and Excel 2019 was used to build a database. No imputation was performed for missing data. Outliers were double-checked with medical records before pursuing data analysis. Access to the electronic database was limited to the study investigators.
Data source location	• Institution: University Hospital Giessen and Marburg, Patienten- Heimversorgung outpatient dialysis center • City/Town/Region: GiessenCountry: Germany
Data accessibility	Repository name: Data on immunogenicity and reactogenicity to COVID-19 vaccination among patients receiving maintenance dialysis Data identification number (permanent identifier, i.e. DOI number): 10.17632/dv2vm47sbm.4 Direct link to the dataset:http://dx.doi.org/10.17632/dv2vm47sbm.4
Related research article	Karakizlis H, Nahrgang C, Strecker K, Chen J, Aly M, Slanina H, Schüttler CG, Esso I, Wolter M, Todorova D, Jessen S, Adamik A, Ronco C, Seeger W, Weimer R, Sester M, Birk HW, Husain-Syed F. Immunogenicity and reactogenicity of homologous mRNA-based and vector-based SARS-CoV-2 vaccine regimens in patients receiving maintenance dialysis. Clin Immunol. 2022 Mar;236:108,961.

## Value of the Data

• Data on SARS-CoV-2-specific cellular and humoral immunity post-vaccination in patients on maintenance dialysis are scarce. We herein provide data on the cellular and humoral immune responses after full SARS-CoV-2 vaccination obtained in a single-center in Germany. These data could be used to compare immunogenicity to COVID-19 vaccination among different populations.
• Researchers and clinicians aiming to understand the determinants of immune responses after full SARS-CoV-2 vaccination can profit from this dataset. The data is also valuable to researchers who would like to compare our results with other studies on COVID-19 vaccine efficacy from other countries, as well as to researchers who want to perform a systematic review and meta-analysis study in the future.
• The dataset and the questionnaire elaborated may be used by other researchers who aim to conduct similar studies in patients on maintenance dialysis.

## 1 Data Description

Patients receiving maintenance dialysis are at increased risk for morbidity and mortality associated with coronavirus disease 2019 (COVID-19) compared with the general population [1,2]. Optimizing the vaccination strategy in this population requires an understanding of the humoral and cellular immune response dynamics to SARS-CoV-2 vaccines, but data on SARS-CoV-2-specific immunity post-vaccination in patients on maintenance dialysis are scarce [3]. The data file shared in the repository contains the raw data on our recently published work [4] evaluating the humoral and cellular immunogenicity and reactogenicity of a homologous mRNA-based and vector-based SARS-CoV-2 vaccine regimen in 60 patients receiving maintenance dialysis (six with a history of COVID-19). Data include information on demographics, comorbidities, dialysis modality and vintage, baseline clinical data, and vaccine regimen. The data file also includes data on the SARS-CoV-2-specific interleukin-2 (IL-2) reactivity and interferon-γ (IFN-γ) reactivity at 4 weeks and 6 weeks following a single dose or a complete homologous dual dose SARS-CoV-2 vaccine regimen, and on the self-reported local and systemic adverse events after the first and second dose using a standardized questionnaire (available at: http://dx.doi.org/10.17632/dv2vm47sbm.4).

## 2 Experimental Design, Materials and Methods

In this study, we investigated the humoral and cellular immunogenicity and reactogenicity of a homologous mRNA-based and vector-based SARS-CoV-2 vaccine regimen in patients receiving thrice-weekly in-center maintenance dialysis (hemodialysis and peritoneal dialysis). At the time of enrollment, the Patienten-Heimversorgung (PHV) outpatient dialysis center located at University Hospital Giessen and Marburg, Giessen, Germany served 84 hemodialysis patients and 5 peritoneal dialysis patients. Patients were approached during their dialysis session for possible participation in the study. Inclusion criteria were: i) recipient of a homologous mRNA-based or a single-dose or homologous dual dose vector-based vaccine regimen (with or without history of COVID-19), and ii) no laboratory evidence of current SARS-CoV-2 infection. All blood samples were obtained prior to dialysis treatment to minimize the risk of leukocyte adhesion to the hemofilter at 4 weeks (T1) and 6 weeks (T2) after complete vaccination, with a tolerance range of ±2 days. The SARS-CoV-2-specific cellular immune response was evaluated using IFN-γ and IL-2 ELISpot (enzyme-linked immune adsorbent spot) assays as recently described [5,6]. The SARS-CoV-2-specific humoral immune response was evaluated using a dot plot array and a chemiluminescent microparticle immunoassay. Detailed information on the methods is provided below.

## 3 ELISpot Method

### 3.1 Isolation of peripheral blood mononuclear cells

Peripheral whole blood samples were collected in sodium citrate tubes and processed within 24–48 h after blood withdrawal to isolate peripheral blood mononuclear cells (PBMC). Whole blood samples were diluted in a ratio of 3:1 with phosphate buffer saline (PBS; Biochrom GmbH, Berlin, Germany) to avoid clotting and then isolated by Ficoll density gradient centrifugation (GE Healthcare Bio-Sciences AB, Uppsala, Sweden). Samples were centrifuged at a rate of 1000 × *g* for 30 min at room temperature with brake off. The PBMC layer was collected and washed three times (twice with PBS and once with AIM-V (Thermo Fisher Scientific Inc., Waltham, United States)). Finally, cells were counted and adjusted to 2 × 10^6^ cells/ml for use in ELISpot assay. The technicians were blinded to the clinical data.

### 3.2 ELISpot assay

The AID/GenID CoV-*i*Spot IFN-γ + IL-2 (ELSP7010; AID GmbH, Strassberg, Germany) detection kit was used for the ELISpot experiments conducted in this study. Briefly, 96-well membrane plates were coated with capture antibodies against human IFN-γ and human IL-2. In each test, complete medium alone and Pokeweed mitogen were used as negative and positive controls, respectively. Furthermore, anti-CD28 was added to each well for co-stimulation.

**Fig. 1:**
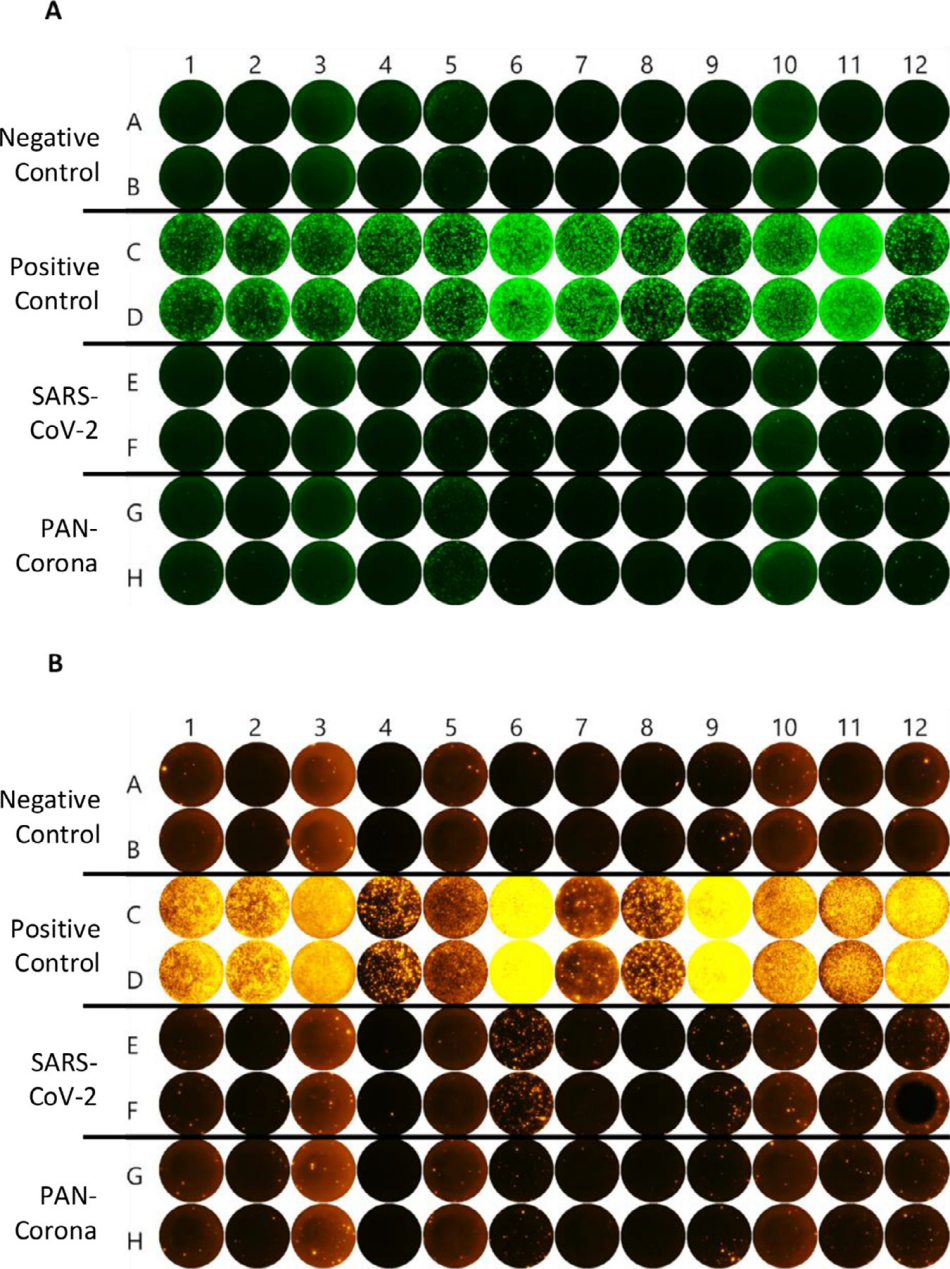
SARS-CoV-2-specific T-cell responses using a multicolor FluoroSpot Immune assay.

For all samples, each control and antigen stimulation was performed in duplicates with 2 × 10^5^ PBMC/well. For stimulation, plates were incubated at 37 °C and 5% CO_2_ for 16–20 hr. After washing steps, the different cytokine fluorospots were detected followed by the addition of fluorescent labelled antibody conjugates which were incubated for one hour. The next day, the spots obtained were automatically counted with the Fluorspot Reader version 8 (AID GmbH, Strassberg, Germany). Fig. 1A and B show representative FluoroSpot wells for each cytokine-producing T cell against antigen S. Any antigen-specific ELISpot test with less than 5 spots/2 × 10^5^ PBMC was considered as negative when assessed in a qualitative manner. The results were considered after subtracting to each well the responses obtained in the respective negative control wells.

The AID/GenID CoV-*i*Spot detection kit includes one SARS-CoV-2 specific peptide pool (SARS-CoV-2 Peptide-Mix) that allows maximal differences to corona viruses other than SARS-CoV-2 (e.g., SARS-CoV-2, SARS-CoV, middle east respiratory syndrome-coronavirus, other corona viruses causing the common cold) and an additional peptide pool with a maximum consensus across different types of the coronaviridae family (PAN—Corona Peptide-Mix).

The majority of the SARS-CoV-2 specific peptides included in the AID/GenID SARS-CoV-2 Peptide-Mix are located in the N-terminal region of the spike protein while the conserved regions included in the PAN—Corona Peptide-Mix represent the C-terminal region. It should be noted that the used antigen peptide pools are not affected by the current key mutations 69–70 del, E484K, N501Y, and D614G.

Representative example results of 12 (columns 1–12) dialysis patients after vaccination, analysed with the AID/GenID CoV-*i*Spot FluoroSpot Assay. Panel A shows the secretion of interferon-γ (green channel) and panel B shows the secretion of interleukin-2 (orange channel). Rows A–B represent the negative control, C–D represent the positive control, E–F represent the stimulation with the SARS-CoV-2 specific Peptide-Mix (SARS-CoV-2), and G–H represent the stimulation with the Coronaviridae family specific PAN—Corona Peptide-Mix (PAN—Corona).

SARS-CoV-2, severe acute coronavirus type-2.

### 3.3 SARS-CoV-2-specific antibodies

SARS-CoV-2-specific antibodies were quantified using plasma from citrated whole blood samples using an immunoglobulin G (IgG) assay coated with a recombinant receptor-binding domain of the SARS-CoV-2 spike protein antigen using an the in-house dot plot array provided by GenID. Antibody levels are expressed in% intensity of gray scale, ranging from 0 to 100 percent black, with an intensity of >16% considered positive and ≤16% considered negative, respectively. Furthermore, SARS-CoV-2-specific antibodies from serum samples against the spike protein and nucleocapsid protein were performed by the Institute of Medical Virology (Giessen, Germany) using antibody chemiluminescent microparticle immunoassay (Anti-S AdviseDx SARS-CoV-2 IgG II and Anti-N Abbott Architect SARS-CoV-2 IgG, Abbott, Chicago, IL, USA). Anti-S levels after infection or vaccination were expressed as AU(arbitrary unit)/ml.

## Ethics Statements

The research was carried out in accordance in accordance with the Declaration of Helsinki. The project was approved by the local Ethical committee (AZ 126/21). Written informed consent was obtained from all participants prior to enrollment in the study.

## CRediT Author Statement

**Hristos Karakizlis:** Conceptualization, Investigation, Resources, Data Curation, Writing - Original Draft, Writing - Review & Editing, Visualization, Supervision, Project administration; **Christian Nahrgang:** Conceptualization, Investigation, Resources, Data Curation, Writing - Original Draft, Writing - Review & Editing, Project administration; **Kevin Strecker:** Conceptualization, Investigation, Resources, Data Curation, Writing - Original Draft, Writing - Review & Editing, Visualization, Project administration; **Jiangping Chen:** Investigation, Writing - Review & Editing, Project administration; **Mostafa Aly:** Formal analysis, Investigation, Data Curation, Writing - Original Draft, Writing - Review & Editing, Visualization, Project administration; **Heiko Slanina:** Conceptualization, Investigation, Resources, Writing - Review & Editing, Project administration; **Christian G. Schüttler:** Conceptualization, Investigation, Resources, Writing - Review & Editing; **Isla Esso:** Investigation, Writing - Review & Editing, Project administration; **Martin Wolter:** Investigation, Writing - Review & Editing, Project administration; **Darina Todorova:** Investigation, Writing - Review & Editing, Project administration; **Sönke Jessen:** Investigation, Resources, Writing - Review & Editing, Project administration; **Andrea Adamik:** Investigation, Writing - Review & Editing, Project administration; **Claudio Ronco:** Investigation, Writing - Review & Editing, Project administration; **Werner Seeger:** Investigation, Resources, Writing - Review & Editing, Project administration; **Rolf Weimer:** Investigation, Writing - Review & Editing, Project administration; **Martina Sester:** Conceptualization, Investigation, Resources, Writing - Review & Editing, Supervision, Project administration; **Horst-Walter Birk:** Conceptualization, Investigation, Resources, Writing - Review & Editing, Supervision, Project administration; **Faeq Husain-Syed:** Conceptualization, Investigation, Resources, Data Curation, Writing - Original Draft, Writing - Review & Editing, Visualization, Supervision, Project administration.

## Declaration of Competing Interest

The authors declare the following financial interests/personal relationships which may be considered as potential competing interests: Kevin Strecker is employee of AID/GenID, the manufacturer of the ELISpot assay. None of the other authors declare any competing interests.

## Data Availability

Data on immunogenicity and reactogenicity to COVID-19 vaccination among patients receiving maintenance dialysis (Original data) (Mendeley Data).
